# "Eight and a half" and "nine syndrome" rare presentation of pontine lesions; case reports and review of literature

**Published:** 2018-10-07

**Authors:** Samira Yadegari, Masoud Aghsaei-Fard, Mohammadreza Akbari, Arash Mirmohammad-Sadeghi

**Affiliations:** 1 Department of Neuro-Ophthalmology and Strabismus, Farabi Eye Hospital, Tehran University of Medical Sciences, Tehran, Iran; 2 Eye Research Center, Farabi Eye Hospital, Tehran University of Medical Sciences, Tehran, Iran

**Keywords:** Eight-and-a-Half Syndrome, Nine Syndrome, Intracerebral Hemorrhage, Demyelination, Neuromyelitis Optica Spectrum Disorder

## Abstract

**Background:** Eight-and-a-half syndrome (EHS) is one-and-a-half syndrome [(conjugated horizontal gaze palsy and internuclear ophthalmoplegia (INO)] plus ipsilateral fascicular seventh cranial nerve palsy. Involvement of lower pontine tegmentum including the abducens nucleus, the ipsilateral medial longitudinal fasciculus (MLF), and the adjacent facial colliculus contribute to the clinical findings of EHS. Recently, nine syndrome with addition of hemiparesis or hemianesthesia to EHS (due to involvement of adjacent corticospinal tract or medial lemniscus) is suggested.

**Methods:** Consecutive patients with presentation of EHS or nine syndrome were reviewed from referral neuro-ophthalmology and strabismus clinics.

**Results:** Three cases of EHS were identified with different etiologies of intracerebral hemorrhage (ICH), demyelination, and neuromyelitis optica spectrum disorder. Moreover, one case of "nine syndrome" due to ICH was described. Brain magnetic resonance imaging (MRI) in all of them revealed lesion in lower tegmentum of pons.

**Conclusion:** Apart from different etiologies, recognition of EHS or nine syndrome allows precise localization of the lesion to lower pontine tegmentum ipsilaterally.

## Introduction

Eight-and-a-half (EHS) syndrome is a rare neuro-ophthalmologic condition which defines as one-and-a-half syndrome plus facial nerve palsy ipsilateral to the side of one eye which has no movement (1.5 + 7 = 8.5). Extension of lesion to adjacent structures such as corticospinal tract, medial lemniscus, or cerebellar peduncle may suggest nine syndrome which is recently proposed (8.5 + 0.5 = 9).^1^^,^^2^ The combination of these clinical findings allows precise localization of the lesion, and therefore could have significant diagnostic value.

## Materials and Methods

Consecutive patients with the diagnosis of EHS or nine syndrome from neuro-ophthalmology and strabismus clinics of a tertiary referral eye hospital were reviewed during the years 2014 to 2016. Complete ophthalmologic and neurologic examination and neuro-imaging were done, and they were followed for at least one year after their presentation.

## Results

**Figure 1:**
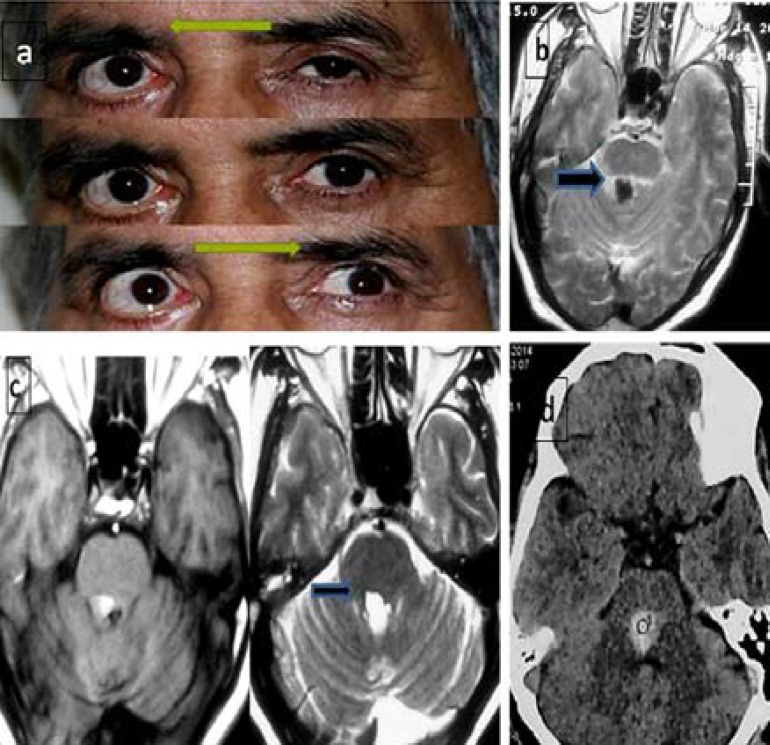
a) Right gaze palsy (upper image) and internuclear ophthalmoplegia (INO) on left gaze (lower image); b) T2 hypointense lesion in dorsal of pons compatible with acute phase of hemorrhage; c) Subacute hemorrhage as hyperintensity in T2 (black arrow) and flair sequences; and d) One year later, the edema resolved and the hemorrhage became hypointense in T2 weighted image.

***Case 1: ***A 54-year-old man presented with sudden onset of diplopia, epiphora in right eye, and facial asymmetry. His past medical history for hypertension, diabetes mellitus, hyperlipidemia, and smoking was unremarkable; but he was addicted to opium. On exam, there was right conjugate gaze palsy and internuclear ophthalmoplegia (INO) on left gaze which presented as no horizontal movements in right eye and adduction limitation in left eye (Figure 1-a). In addition, he had right-lower-motor-neuron-type facial paresis. Other neurologic exams were within normal limits. Brain magnetic resonance imaging (MRI) revealed hypointensity signal in posterior part of pons in T2 and flair sequences compatible with acute phase of intracerebral hemorrhage (ICH) surrounded by edema (Figure 1-b). 

One month later, the signal changed to hyperintensity in T1, T2, and flair sequences (subacute phase of hemorrhage, Figure 1-c), and peripheral edema resolved. On one-year follow up (Figure 1-d), he had no improvement neither in eye movement nor facial paresis. He was referred for strabismus surgery.

***Case 2:*** A 47-year-old woman with history of hypertension and hyperlipidemia from 7 years ago, presented with nausea and vomiting, diplopia, and left-side weakness. Neurologic exams showed right gaze palsy, INO on left gaze, right facial paresis, and left hemiparesis and hemianesthesia. Brain computed tomography (CT) scan revealed ICH in the right pons involving area of the abducens nucleus, adjacent medial longitudinal fasciculus (MLF), and facial colliculus, extending to the ipsilateral medial lemniscus and corticospinal tract. She was managed medically. On 2-year follow up, facial paresis and adduction of both eyes showed some improvement, but right eye abduction was unchanged.

***Case 3:*** A 29-year-old single woman presented with diplopia and facial asymmetry since 2 weeks ago. She had no history of any underlying disease. On exam, she had right gaze palsy, INO on left gaze, and peripheral-type right facial palsy. Other neurologic exams were unremarkable. Brain MRI revealed multiple periventricular T2 and flair hypersignal plaques, and one in right posterior of pons, ventral to fourth ventricle with no gadolinium enhancement. Vasculitic tests were negative. One month after 5 grams methylprednisolone pulse, her symptoms completely resolved. Low-frequency interferon beta-1a was started for her regarding high burden of the lesions. She had no diplopia after one year.

***Case 4:*** A 34-year-old man developed intractable nausea and vomiting for one week. He underwent endoscopy which was unremarkable. He concurrently was bothered by diplopia and loss of taste of right tongue. He had right gaze palsy, INO on left gaze, and right peripheral type facial palsy. Other neurologic exams were normal. Brain MRI was unremarkable except for one hypersignal lesion in posterior of right pons in flair and T2 weighted images. Cervical and thoracic MRI was unremarkable as neuromyelitis optica (NMO) antibody and vasculitis test. Visual evoked potential showed increased P100 latencies in both eyes (125 and 130 milliseconds). After 5 grams of pulse steroid, diplopia and loss of taste was improved, but he had mild abduction limitation of right eye. Oral prednisolone was started with impression of seronegative NMO spectrum disorder.

## Discussion

One-and-a-half syndrome is combination of horizontal conjugate gaze palsy [due to involvement of paramedian pontine reticular formation (PPRF) or/and sixth nerve nucleus], and INO because of ipsilateral medial longitudinal fascicle (MLF) involvement. It represents as limited horizontal movements of both eyes, except for abduction of one eye. Involvement of facial colliculus which is the fascicle of seventh cranial nerve that turns around sixth nerve nucleus may cause EHS.

Until now, 18 cases of EHS have been reported and the etiology in most of them was ischemic infarction in dorsal tegmentum of pons. Other etiologies included infection in one (tuberculoma),^3^ ICH in one,^2^ giant cell arteritis in one^,^^4^ and demyelination compatible with multiple sclerosis in three patients.^5^^,^^7^ In our cases, case 1, 3, and 4 were EHS with ICH, demyelination, and NMO spectrum disorder etiologies.

Nine syndrome was reported previously only in two cases,^1^^,^^2^ and both of them were due to pontine infarction. The etiology in our case (case 2) was pontine ICH. At least, one-year follow-up of our 2 patients with ICH (case 1 and 2) revealed that their clinical presentations were a consequent of direct tissue damage rather than edema secondary to hemorrhage.

## Conclusion

The involvement of abducens nucleus, ipsilateral MLF, and facial nucleus/fascicles in the lower pontine tegmentum have clinical presentations of EHS. Extension of lesion to cerebral peduncle or medial lemniscus represents nine syndrome. The corresponding lesion may have different etiologies, but recognition of these syndromes allows precise localization of the lesion to lower pontine tegmentum ipsilaterally.

## Notes:

**How to cite this article:** Yadegari S, Aghsaei-Fard M, Akbari M, Mirmohammad-Sadeghi A. "Eight and a half" and "nine syndrome" rare presentation of pontine lesions; case reports and review of literature. Iran J Neurol 2018; 17(4): 189-91.

## References

[1] Rosini F, Pretegiani E, Guideri F, Cerase A, Rufa A (2013). Eight and a half syndrome with hemiparesis and hemihypesthesia: The nine syndrome?. J Stroke Cerebrovasc Dis.

[2] Mahale RR, Mehta A, John AA, Javali M, Abbas MM, Rangasetty S (2015). "Nine" syndrome: A new neuro-ophthalmologic syndrome: Report of two cases. Ann Indian Acad Neurol.

[3] van Torn R, Schoeman JF, Donald PR (2006). Brainstem tuberculoma presenting as eight-and-a-half syndrome. Eur J Paediatr Neurol.

[4] Eggenberger E (1998). Eight-and-a-half syndrome: One-and-a-half syndrome plus cranial nerve VII palsy. J Neuroophthalmol.

[5] Skaat A, Huna-Baron R (2012). Eight-and-a-half syndrome: A rare pontine neuro-ophthalmologic syndrome. Arch Neurol.

[6] Mortzos P, Nordling MM, Sorensen TL (2014). Eight-and-a-half syndrome as presenting sign of childhood multiple sclerosis. J AAPOS.

[7] Wanono R, Daelman L, Maarouf A, Caucheteux N, Chaunu MP, Tourbah A (2014). Eight and a half plus syndrome as a first presentation of multiple sclerosis. Rev Neurol (Paris).

